# Predictors and memory consequences of dating decisions in a dating app-analogue study

**DOI:** 10.1017/ehs.2024.22

**Published:** 2024-05-20

**Authors:** Yikang Zhang, Pekka Santtila

**Affiliations:** 1 Faculty of Psychology and Neuroscience, Maastricht University, Maastricht, the Netherlands; 2 New York University Shanghai, Shanghai, China.

**Keywords:** Online dating, disgust, mate value, sexual strategy, memory

## Abstract

With the rise of dating apps, people have access to a vast pool of potential partners at their fingertips. The present study examined how various factors would predict an individual's dating decisions in a dating app-analogue study. Participants (*N* = 269) first completed some trait measures and then a mock-dating task in which they judged the attractiveness of a series of targets and then decided whether to match with the target or not. Their memories for the targets were tested on the second day. People who were more (vs. less) short-term oriented were more likely to match with short-term-oriented targets. Moral disgust and sexual disgust negatively predicted the matching with short-term-oriented targets. Contrary to our hypothesis, we did not find support that people with higher (vs. lower) pathogen disgust sensitivity would selectively match with more attractive targets. Exploratory analyses showed that people who were more (vs. less) short-term oriented, more (vs. less) sexually attractive, or had higher (v. lower) mate value, were more likely to match with targets they considered as attractive. Finally, people have better memories of the faces they chose to match than to not match. Implications for mating research and limitations are discussed.

## Graphical abstract

**Figure:**
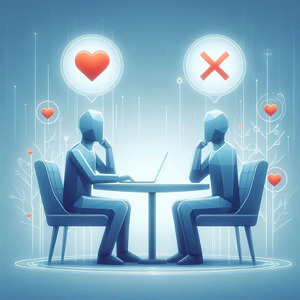

Social media summary: This study investigated factors influencing dating decisions. Those more short-term oriented were likelier to match with similar partners. Short-term oriented, sexually attractive and high mate value individuals were likelier to match with targets they found attractive.

## Introduction

For a sexually reproductive species like human, mating plays a central role in individuals’ fitness, resulting in us being equipped with various evolved psychological mechanisms to tackle mate selection. A wealth of research in this area has shown that individuals’ mate choice decisions are influenced by their own attributes, such as mate value (Arnocky, 2018; Buss, 2003), their relationship goals (e.g. short-term vs. long-term, Quist et al., 2012; Sacco et al., 2009; Zhang et al., 2022), and the broader social and cultural context (e.g. sex ratio, Stone et al., 2007; Walter et al., 2021). With the rise of online dating apps such as Tinder or Grindr (and their Chinese counterparts such as Tantan and Blued), the way we choose potential partners has changed. Instead of face-to-face interactions within one's local community, individuals now have access to a vast pool of potential partners across nations at their fingertips. Moreover, given how the apps have been constructed, online dating prioritises (curated) physical appearance over other features more than traditional dating contexts. It has been shown that online dating brings along various psychosocial risks such as negative body image, aggression, as well as changes in sexual behaviour in the form of more casual sexual encounters and relationships under the effects of alcohol and other drugs (Castro & Barrada, 2020). Also, decreases in general wellbeing have been reported (Zervoulis et al., 2020). Understanding the decision-making processes in online dating could be important for promoting the healthy use of online dating apps. As of now, the online dating decision-making process from an evolutionary perspective is still understudied. Ranzini et al. (2022) designed a mock dating app to study assortative mating regarding race, ethnicity and education in the Dutch context and found support for assortative mating in terms of education and ethnicity. De La Mare and Lee (2023) similarly used a mock dating task to examine assortative mating regarding the big-five personality traits. Building on their approach, in the present study, we examined how individuals varying in mate value, relationship goals and other attributes choose their potential mates in a dating app-analogue experiment. In the following section, we briefly review the literature related to mate choice and introduce the aims and hypotheses of the present study.

### Sexual strategies

According to the sexual strategies theory (Buss, 1998; Buss & Schmitt, 2017), individuals can have different sexual strategies ranging from a preference for short-term casual sex to one for long-term relationships. The adoption of these strategies is influenced by evolutionary constraints in the past such as the sex difference in obligatory parental investment (Trivers, 1972), individual attributes like mate value, attractiveness and disgust sensitivity, and contextual variables such as the sex ratio of the local mating pool and social norms. More relevant to the present study, research has shown that sexual strategies influence people's mating preferences regarding their potential partners. Compared with people who are less oriented towards short-term mating, people with greater short-term mating orientation showed greater sensitivity (Sacco et al., 2009) and preference (Quist et al., 2012) for symmetrical faces, a characteristic associated with perceived attractiveness, and placed greater importance on mate's physical attractiveness (Simpson & Gangestad, 1992; Zhang et al., 2022). Further, research on mobile dating app use showed that people who pursue short-term (vs. long-term) mating are heavier users of dating apps (Botnen et al., 2018; Konings et al., 2022).

Although long-term mating strategy is negatively correlated with short-term strategy at the group level, they are not the opposite ends of a single dimension. The pursuit of short-term mating does not entail no or low interest in long-term mating (Jackson & Kirkpatrick, 2007) and there are cases where these two converge. For example, it has been found that consistent with the parental investment theory (Trivers, 1972), men, compared with women, have a greater preference for short-term mating. However, the two sexes do not show large differences in the pursuit of long-term dating (Jackson & Kirkpatrick, 2007; Zhang et al., 2022). Therefore, when examining the effect of mating orientation on mate choice, it can be of added value if we include both measures of short-term mating and long-term mating (Zhang et al., 2022). Moreover, it has been found that assortative mating (Vandenberg, 1972), a phenomenon where individuals tend to mate with partners who are similar to themselves in socioeconomic status, education (Krzyżanowska & Mascie-Taylor, 2014), race (Ranzini et al., 2022) or personality (Glicksohn & Golan, 2001; Luo, 2017), extends to sexual strategies. People who pursue either short-term or long-term strategies would also prefer potential partners who share their mating orientation (Zhang et al., 2022).

Taken together, we hypothesised that people with stronger short-term (vs. long-term) mating orientation would be more likely to selectively match with attractive individuals (H1). In addition, we expected that short-term (vs. long-term) oriented individuals would match with targets with short-term mating orientation more while the opposite pattern holds for targets with long-term mating orientation (H2).

### (Self-perceived) mate value

Mate value refers to an individual's perceived desirability as a potential mate, based on factors such as physical appearance, social status, resources and personality (Buss, 2003; Edlund & Sagarin, 2014). Research has shown that mate value impacts mate choice in various ways (e.g. Arnocky, 2018; Conroy-Beam et al., 2016; Edlund & Sagarin, 2010; Regan, 1998). Consistent with the more general pattern of assortative mating, men who perceive themselves as having higher mate value also tend to look for high-mate-value partners (Arnocky, 2018) and have more attractive partners (Udry & Eckland, 1984). Other research also found that women's self-perceived mate value and their partner's mate value are positively correlated (Miner et al., 2009). Therefore, we hypothesised that people who perceive themselves as having high mate value would be more likely to selectively match with attractive individuals than their counterparts who have low self-perceived mate value (H3).

### Disgust sensitivity

The emotion of disgust has been proposed as a behavioural avoidance mechanism (Tybur et al., 2013). According to this framework, there are three types of disgust based on their elicitors as well as motivational and behavioural consequences: pathogen (related to avoiding disease), sexual (related to avoiding harmful sexual encounters) and moral (related to coordinating social interactions) disgust (Tybur et al., 2009, 2013). Mate selection involves identifying valuable mates, and avoiding contagion by pathogens, and is embedded in larger social and cultural contexts. These aspects of mating are associated with disgust sensitivity. For example, research has shown that high pathogen disgust sensitivity is related to a greater preference for sexually dimorphic features, supposedly indicators of health (e.g. DeBruine et al., 2010; Jones et al., 2013), and greater emphasis on the physical attractiveness of potential mates (e.g. Park et al., 2012; Zhang et al., 2022; for a review, see Beall, 2021). However, a recent registered report failed to find support for the link between pathogen disgust and the preference for sexually dimorphic or symmetric faces in both men and women (Tybur, Fan et al., 2022), casting doubt on the robustness of the previous findings and in extension the link between pathogen disgust and preference for attractiveness. The null findings from Tybur, Fan et al. (2022) also raise the question of whether support for the effect can be found in other paradigms, such as mock dating. Sexual disgust, on the other hand, has a robust negative association with the preference for short-term mating across societies (Al-Shawaf et al., 2015, 2019; O'Shea et al., 2019; Zhang et al., 2022). As for moral disgust, although it does not consistently predict short-term strategy (Hlay et al., 2022), it has been shown to be positively correlated with long-term mating orientation (Zhang et al., 2022). Moral disgust has been argued to be associated with norm compliance (Zhang et al., 2022). For example, people with a higher (vs. lower) moral disgust sensitivity also show a higher justice sensitivity, being more sensitive to others’ and one's own moral transgressions (Bondü & Richter, 2016). Given that long-term mating is perceived to be more socially approved than short-term mating even though the latter is not always disapproved (Zhang et al., 2022), we predict that moral disgust will be positively associated with a preference for long-term-oriented partners as well.

Taking the above together, we hypothesised that people with higher pathogen disgust sensitivity would be more likely to restrict their matches to attractive individuals than their counterparts who have low pathogen disgust sensitivity (H4). People with higher sexual disgust sensitivity would be less likely to match with individuals with short-term mating orientation than their counterparts who have low sexual disgust sensitivity (H5). Finally, we predicted that people with higher moral disgust sensitivity would be more likely to match with individuals with long-term mating orientation than their counterparts who have low moral disgust sensitivity (H6).

### Testing directed forgetting as a consequence of mating decisions

Directed forgetting is a robust phenomenon in psychology (e.g. Basden et al., 1993; Hauswald & Kissler, 2008; Thompson et al., 2011). In typical directed forgetting studies, people first study items (often words but also pictures) for a later memory test. Some items are to-be-remembered items that are cued with a remembering instruction, whereas other items are to-be-forgotten items with a forgetting cue. In the end, participants are tested for the memory of the to-be-remembered items but also the to-be-forgotten ones. In short, compared with to-be-remembered stimuli, people tend to have worse memories for presented stimuli that are instructed to be forgotten. Moreover, it has been proved that the effect of directed forgetting extends to memory for faces, with people having lower memory accuracy for to-be-forgotten faces (Corenblum et al., 2020; Metzger, 2011). The research paradigm of directed forgetting shares many similarities with swapping left or right on dating apps. In both cases, individuals receive either internal (dating decision) or external cues (directed forgetting) that the stimuli are important to remember or not. Therefore, it is reasonable to assume that people would have a better memory for the datable faces compared with the not-datable faces. Addressing the role memory plays in dating decisions may further clarify differences in perceived mating pools among people differing on various traits (Crosby et al., 2021). We, therefore, included a recognition task to examine whether people's memory for the targets would be influenced by their matching decisions earlier, with people having worse memories for the faces they rejected than the faces they matched after controlling for the attractiveness of the targets (H7). Below, we briefly summarise the design of the current study.

## The current study

In the current study, participants first completed a set of trait measures assessing their sexual strategies, mate value and disgust sensitivity. Then they played a mock dating game where they were presented with a series of photos of the sex to which they were attracted. These photos varied in attractiveness judged by an independent group and were randomly described as either pursuing a long-term or a short-term relationship. Participants first rated how attractive each photo was and then decided if they chose to match with this person. One day after the dating game, participants completed a recognition task assessing their memory for the faces as well as their decisions associated with the faces.

### Method

### Participants

The study acquired ethical approval at the Ethics Review Committee Psychology and Neuroscience (reference ERCPN 264_25_02_2023) before data collection. We recruited online workers from the crowdsourcing platform Prolific (https://www.prolific.co/). To participate in the study, individuals needed to be between 18 and 30 years old. The reason to include an upper age limit was that the photos in the current study were of university students and that age differences influence people's mating decisions. For example, women are less inclined to express interest in men younger than themselves (Conway et al., 2015). Participants received £3 as compensation for participation in the whole study and £1.8 if they only completed the mock-dating task but not the memory test.

#### A priori power analysis

Metzger (2011) reported that the effects of directed forgetting on the recognition memory of faces were from *η*^2^_p_ = 0.25 to *η*^2^_p_ = 0.56. Corenblum et al. (2020) reported relatively smaller effects from *η*^2^_p_ = 0.16 to *η*^2^_p_ = 0.29. Taking into consideration that the current study does not manipulate the cue of forget/remember explicitly and that the cues will not be assigned randomly, our smallest effect size of interest (SESOI) for the directed forgetting effect is *η*^2^_p_ = 0.10. *A priori* power analysis for within-subject ANOVA showed that with *α* = 0.05 and 1 − *β* = 0.95, a sample of 20 could reliably detect an effect of *η*^2^_p_ = 0.10. However, as we are mainly interested in exploring the relationship between dating decisions and individual differences, and the effects between relevant individual differences and mating preferences range from |*r*| = 0.20 to 0.48 in Zhang et al. (2022), we therefore simulated a dataset to perform a simulation-based power analysis for generalised linear mixed models (GLMM).

Our SESOI was set to be *r* = 0.10, which translates to a log odds ratio of 0.365 (https://www.escal.site/). We, therefore, expect that for example, a one-standard deviation increase of pathogen disgust sensitivity will lead to an increase of 0.365 in the regression coefficient of Attractiveness (standardised) per H4. Since we set the SESOI to be the same across H1–H6, being a log odds ratio of 0.365, we choose one specific model testing H4 for the simulation-based power analysis using the R package mixedpower (Kumle et al., 2021). Based on the hypothesised effect, we composed a simulated dataset with participants varying in pathogen disgust and target photos varying in attractiveness. Then we calculated the log-odds of each matching decision based on the target's attributes (e.g. target's attractiveness) and participants’ traits (i.e. pathogen disgust sensitivity), which then were used to generate the responses (Match vs. Not match). Results showed that the estimated parameters of the intercept-only model (e.g. intercept and random effects, see https://osf.io/pgshk?view_only=4941cc2d1f534c7a813260b5be19f973 for the output) were similar to those of the pilot data (see below), supporting the validity of the simulated data. owing to the constraints of the package, when performing power analyses, we only had a random intercept for participants but not for target faces. For fix effects, we included pathogen disgust sensitivity, target attractiveness, and their interaction term. The results showed that a sample of 80 could have a power of 0.98 to detect the hypothesised effect while a sample of 270 could have a power of >0.99 to detect the hypothesised effect (critical value = 1.96, *n*_sims_ = 1000). Based on the power simulation, we decided to recruit 270 participants to reach robust conclusions.

A total of 272 participants completed the mock-dating task, of whom three reported not taking the task seriously (seriousness < midpoint 3) and 29 participants matched with either all or none of the targets. Given that self-reported seriousness is associated with data quality (Aust et al., 2013), individuals reporting being not serious when completing the study (seriousness < 3) were excluded from analyses. Deviating from stage 1 exclusion criteria, we did not exclude participants who indiscriminately matched or did not match with all targets. The reason is that men were more likely to match with all targets while women were more likely to not match with any targets, which supports the validity of their matching decisions. The final sample therefore consisted of 269 participants (*n*_male_ = 131, *n*_female_ = 137, *n*_no disclosure_ = 1; M_age_ = 25.2; SD_age_ = 3.1).

### Materials

#### Facial stimuli

A set of 120 colour full-frontal-view photographs of males (*n* = 60) and females (*n* = 60) with natural expressions were randomly selected from the Oslo Face Database (OFD; Chelnokova et al., 2014). Among the 60 male or female faces, a random subset of 40 photos were presented during the matching session (hereafter referred to as Old) and the remaining 20 photos were only presented in the recognition phase as fillers (hereafter referred to as New). The faces from the OFD database contain ratings of attractiveness, trustworthiness and dominance on seven-point scales from independent raters. We, therefore, performed a series of two-way ANOVAs to examine whether there were group differences. The results showed that for attractiveness, we did not find support for a main effect of sex, *F* (1, 116) = 0.21, *p* = 0.649, a main effect of Old vs. New, *F* (1, 116) = 0.67, *p* = 0.414, or the interaction, *F* (1, 116) = 0.30, *p* = 0.584. For trustworthiness, we found support for a main effect of sex, *F* (1, 116) = 5.58, *p* = 0.020, with male faces being judged as slightly more trustworthy than female faces, but not for a main effect of Old vs. New, *F* (1, 116) = 0.34, *p* = 0.561, or the interaction, *F* (1, 116) = 0.05, *p* = 0.832. For dominance ratings, we did not find support for a main effect of sex, *F* (1, 116) = 0.16, *p* = 0.694, a main effect of Old vs. New, *F* (1, 116) = 0.00, *p* = 0.982, or the interaction, *F* (1, 116) = 0.04, *p* = 0.846. The descriptive results are in Table 1. The code can be accessed at https://osf.io/t4xue?view_only=4941cc2d1f534c7a813260b5be19f973.

**Table 1.** Descriptive results for the face stimuli

		Attractiveness		Trustworthiness		Dominance	
		Mean	SD	Mean	SD	Mean	SD
Old	Female	4.02	0.97	5.54	0.71	4.10	0.77
Old	Male	4.29	1.18	5.93	0.93	4.18	0.74
New	Female	4.22	0.82	5.66	0.58	4.13	0.70
New	Male	4.27	0.95	5.98	0.69	4.16	0.90

To further confirm the validity of the mock dating task, we recruited 30 (*n*_male_ = 9, *n*_female_ = 19, *n*_non-disclosure_ = 2) participants to complete the said task. On average, participants considered the task as more or less similar to their dating app experience (mean, *M* = 2.93, standard deviation, SD = 0.78, on a five-point scale from 1 = *not at all* similar to 5 = *very much*). On average, participants matched with 6.83 (SD = 7.67) out of the 40 targets (17.1%). An intercept-only generalised linear mixed model for the matching decision (binomial response: Match vs. Not match) with random intercepts for participant ID and target ID showed that the estimate for the intercept *B* was −2.71, 95% CI [−3.48, −1.94], which was very similar to the results from Ranzini et al. (2022). Intra-class correlations (ICC) of the model showed considerable correlations for participant clusters (ICC = 0.365) and target clusters (ICC = 0.247). Based on these results, we concluded that the task achieved sufficient validity.

#### Self-perceived mate value

Self-perceived mate value (SPMV) was measured with the four-item Mate Value scale (Edlund & Sagarin, 2014). Participants were first presented with an instruction explaining different aspects of mates value such as physical attractiveness, health, personality traits and resources. Then they responded to items such as ‘Overall, how would you rate your level of desirability as a partner on the following scale?’ on a scale from 1 = *Extremely undesirable* to 7 = *Extremely desirable* (*Cronbach's α* = 0.90, McDonald's *ω* = 0.91). In addition, we included a scale measuring a specific aspect of perceived mate value: sexual attractiveness. It was measured with the six-item Self-Perceived Sexual Attractiveness scale, with a higher score indicating greater perceived sexual attractiveness (Amos & McCabe, 2015). Participants responded to items such as ‘I believe I can attract sexual partners’ on a scale from 1 = *Strongly disagree* to 7 = *Strongly agree* (*Cronbach's α* = 0.96, McDonald's *ω* = 0.97).

#### Short-term mating strategy

Short-term mating strategy was operationalised by the nine-point-scale version of the Revised Sociosexual Orientation Inventory (SOI-R; Penke & Asendorpf, 2008). The SOI-R contains three subscales measuring sociosexual behaviour (e.g. ‘With how many different partners have you had sex within the past 12 months?’; Cronbach's *α* = 0.94, McDonald's *ω* = 0.94), attitude (e.g. ‘I can imagine myself being comfortable and enjoying “casual” sex with different partners’; Cronbach's *α* = 0.80, McDonald's *ω* = 0.81) and desire (e.g. ‘How often do you experience sexual arousal when you are in contact with someone you are not in a committed romantic relationship with?’; Cronbach's *α* = 0.90, McDonald's *ω* = 0.90). We collapsed the three subscales together to calculate the mean of SOI-R in subsequent analyses, with a higher score indicating a stronger inclination towards short-term mating.

#### Long-term mating strategy

Long-term mating orientation (LTMO) was measured by six items by Jackson and Kirkpatrick (2007), with a higher score indicating a greater desire to be in a long-term relationship. Items from LTMO were modified by removing the word ‘special’ from the items (e.g. ‘interested in maintaining a long-term romantic relationship with someone special’) to separate the willingness to be in a long-term relationship from the preference for someone special. Participants responded to the items on a seven-point Likert scale from 1, *highly disagree* to 7, *highly agree* (Cronbach's *α* = 0.92, McDonald's *ω* = 0.94).

#### Disgust sensitivity

The Three-Domain Disgust scale (Tybur et al., 2009) was used to measure types of disgust sensitivity, with a higher value indicating greater sensitivity to relevant stimuli. A minor adjustment was made to item 20 (‘having anal sex with someone of the opposite sex’ to ‘having anal sex with a sexual partner’). Participants rated how disgusted they would feel if they were in those situations on a seven-point Likert Scale from 1, *not at all disgusted* to 7, *highly disgusted* (pathogen disgust – Cronbach's *α* = 0.79, McDonald's *ω* = 0.84; moral disgust – Cronbach's *α* = 0.81, McDonald's *ω* = 0.86; sexual disgust – Cronbach's *α* = 0.77, McDonald's *ω* = 0.83).

### Procedures

#### Matching phase

Participants first read the information letter where they were introduced to the aims and tasks of the study as well as the cost and benefits. After giving informed consent, participants first answered a set of demographic questions including age, sex, sexual orientation and relationship status. Then, participants finished the self-perceived sexual attractiveness, SPMV, Three-Domain Disgust scale, SOI-R, and LTMO in random order. Two attention check questions (e.g. ‘This is an attention check, please select 3 for this question’) were embedded in the scales. The order of the items within the scales was also randomised.

After the trait measures, participants were instructed to imagine that they were in a dating scenario and view a series of profile photos of potential dates. Participants attracted to females were presented with the female photo sets. Participants attracted to males were presented with the male photo sets. For people who were attracted to both sexes, we instructed them to select the sex that they found more attractive in general and presented them with photos of that sex. If, however, they indicated that they were attracted to both males and females equally, they were directed to the end of the study and asked to return the task. Each photo was presented for 4 s in a randomised order. Moreover, we manipulated the mating orientation of the targets so that each target photo had a 50% chance of being paired with a long-term orientation statement (e.g. looking for a long-term partner) or a short-term orientation statement (i.e. ‘looking for short-term fun’). Then participants rated the level of attractiveness of that photo on a seven-point scale (‘To what extent do you think this person is attractive in comparison with others?’ – 1 = *Not at all* to 7 = *Extremely*) and whether or not they would match with this person (yes or no). After the mock dating task, participants completed a set of funneling questions including (1) what decisions they are asked to make in the game; (2) how many profiles they see; and (3) how similar is the game to their online dating experience. Then they received their compensation for this session and were reminded to sign up for the next session on the second day.

#### Testing phase

The day after the matching task, participants signed up and completed the recognition task, in which they were presented with a set of 60 photos, consisting of 40 old photos and 20 new photos. For each photo, participants needed to first indicate whether the photo is old or new (‘Have you seen this person in the dating phase?’ – yes/no), and then indicate if they chose to match with this person or not if they recognised the photo as old (‘Did you choose to match with this person or not?’ – not match/Match). Then they first were notified that the answers to the following funnelling questions would not affect their compensation and then completed a set of funnelling questions asking about their seriousness when completing the study (1 = not serious at all, 5 = very serious) and the aim of the study (open-ended question). Finally, they were debriefed and received their compensation for the second session.

### Data analysis overview

All data analyses were carried out in R (version 4. 2.2, R Core Team, 2021). We used (generalised) linear mixed models with the lme4 package (Bates et al., 2014) to test our hypotheses H1–H7. For all models, we included random intercepts for participant ID and photo ID to control the non-independence of the data. All continuous measures concerning participants’ individual differences and targets’ differences were standardised when entered into the models.

In the results section, we first present descriptive results, followed by planned analyses testing H1–H7. In the planned analyses, we used the normative ratings of target attractiveness from OFD (hereafter simply referred to as target attractiveness) to test our hypotheses. Given that there are sex differences regarding sociosexuality and sexual disgust (e.g. Zhang et al., 2022), to control the spurious associations caused by sex, we also included sex and the interaction term between sex and attractiveness or mating goal manipulation depending on the hypotheses to control the effect of sex for hypotheses H1, H2 and H5. Deviating from the analyses plan reported in Stage 1, we also controlled sex in the analyses testing H4, given that male and female participants also showed considerable difference in pathogen disgust sensitivity in our data (see descriptive results below). Then in the section *Exploratory Analyses,* for hypotheses concerning target attractiveness, we further ran analyses with the ratings from the participant in the mock-dating task (hereafter simply referred to as participant-rated target attractiveness) and reported these analyses. We return later to consider the strength and weaknesses of these two approaches and the implications of the results in the Discussion section.

## Results

### Descriptive results

As shown in Table 2, consistent with previous research, short-term mating orientation, and long-term mating orientation had a moderate negative correlation. Further, short-term mating orientation was negatively associated with sexual disgust sensitivity, and long-term mating orientation was positively associated with moral disgust sensitivity. Self-perceived mate value and sexual attractiveness correlated with each other highly. Yet they showed distinct association patterns with sexual strategy: sexual attractiveness was positively associated with short-term mating while mate value was positively associated with long-term mating. Types of disgust sensitivity showed weak-to-moderate positive correlations with one another. Regarding sex differences, female participants on average had lower short-term mating orientation and higher long-term mating orientation than male participants. Female participants also reported higher sexual and pathogen disgust sensitivity than male participants.

**Table 2.** Means, standard deviations, and correlations between trait measures

Variable	*M* (SD)_female_	*M* (SD)_male_	1	2	3	4	5	6	7
1. SOI_R	−0.96 (1.98)	1.00 (2.52)**		−0.29**	0.21**	0.08	−0.14*	−0.40**	0.06
				[−0.39, −0.17]	[0.09, 0.32]	[−0.04, 0.20]	[−0.26, −0.02]	[−0.49, −0.29]	[−0.06, 0.18]
2. LTMO	6.18 (0.98)	5.86 (1.14)*	−0.32**		0.11	0.14*	0.16*	-0.06	0.00
			[−0.42, −0.21]		[−0.01, 0.23]	[0.02, 0.25]	[0.04, 0.27]	[−0.18, 0.06]	[−0.12, 0.13]
3. SPSA	4.69 (1.50)	4.52 (1.28)	0.17**	0.12		0.70**	0.01	−0.17**	0.05
			[0.05, 0.28]	[−0.00, 0.23]		[0.64, 0.76]	[−0.11, 0.13]	[−0.29, −0.05]	[−0.07, 0.17]
4. Mate Value	4.61 (1.22)	4.50 (1.00)	0.05	0.14*	0.70**		0.07	-0.10	0.01
			[−0.07, 0.17]	[0.02, 0.26]	[0.64, 0.76]		[−0.05, 0.19]	[−0.22, 0.02]	[−0.11, 0.13]
5. Moral disgust	4.25 (0.96)	4.16 (0.93)	−0.15*	0.16**	0.01	0.07		0.13*	0.26**
			[−0.26, −0.03]	[0.04, 0.27]	[−0.11, 0.13]	[−0.05, 0.18]		[0.01, 0.25]	[0.14, 0.36]
6. Sexual disgust	2.77 (1.06)	1.99 (1.13)**	−0.48**	−0.01	−0.14*	−0.08	0.14*		0.26**
			[−0.56, −0.38]	[−0.12, 0.11]	[−0.26, −0.02]	[−0.20, 0.04]	[0.02, 0.26]		[0.15, 0.37]
7. Pathogen disgust	4.11 (0.98)	3.58 (0.98)**	−0.05	0.04	0.06	0.02	0.26**	0.33**	
			[−0.17, 0.07]	[−0.08, 0.16]	[−0.06, 0.18]	[−0.10, 0.14]	[0.15, 0.37]	[0.22, 0.43]	

*Note:* SOI-R and LTMO refer to sociosexual orientation and long-term mating orientation respectively, with a higher value indicating a more unrestricted sociosexual orientation or greater long-term mating orientation. SPSA refers to self-perceived sexual attractiveness, with a higher value indicating higher perceived sexual attractiveness.

*M* and SD are used to represent mean and standard deviation, respectively. Values in square brackets indicate the 95% confidence interval for each correlation.

Below the diagonal are the bivariate correlations between variables and above the diagonal are the partial correlations controlling for sex.

* *p* < 0.05; ** *p* < 0.01.

As for the mock-dating task, on average, male participants chose to match with more targets (*M* = 10.66, SD = 7.96) than female participants (*M* = 5.51, SD = 5.48) in the mock-dating task, *F* (1, 265) = 38.05, *p* < 0.001, *η*^2^ = 0.13.

### Hypothesis testing

#### Sexual strategies

To test if people with stronger short-term mating orientation will be more likely to selectively match with attractive individuals (H1), we ran a GLMM with Match decision as the dependent variable, sociosexuality, target attractiveness, sex, the interaction term between sociosexuality and target attractiveness, and the interaction term between sex and target attractiveness as fixed effects. For random effects, we included random intercepts for participants and targets. Results showed that both sociosexuality (*B* = 0.44, SE = 0.11, *p* < 0.001) and target attractiveness (*B* = 0.74, SE = 0.15, *p* < 0.001) positively predicted matching decision. However, the interaction between sociosexuality and target attractiveness was not statistically significant (*B* = 0.02, SE = 0.04, *p* = 0.530). Therefore, we did not find support for H1 based on the planned analysis. Female participants, compared with male participants, were less likely to choose to match (*B* = −0.58, SE = 0.27, *p* = 0.033), and were less influenced by target attractiveness (*B* = −0.25, SE = 0.11, *p* = 0.016).

To test if there is assortative mating with regards to sexual strategies (H2), we ran two models predicting matching decisions with target orientation, sexual strategy (short-term or long-term), and their interaction terms as fixed effects. For the sociosexuality model, we also included Sex and the interaction between Sex and target orientation. The results are presented in Table 3. People with a stronger short-term mating orientation were more likely to choose to match while people with a stronger long-term mating orientation were less likely to do so. People, on average, matched more with targets with a long-term orientation. Most importantly, we found support for the interactions between participants’ sexual strategy and targets’ orientation. People with a stronger short-term mating orientation were less likely to match a long-term-oriented target than their counterparts with a less strong short-term orientation, as indicated by the negative interaction between sociosexuality and target orientation. The opposite holds for long-term orientation. People with a stronger long-term mating orientation were more likely to match a long-term-oriented target than their counterparts with a less strong long-term orientation. H2 therefore received support from our data.

**Table 3.** Assortative mating in sexual strategy

	SOI-R model				LTMO model		
	*B*	SE	*p*		*B*	SE	*p*
**Fixed effects**							
Intercept	−2.28	0.25	<0.001	Intercept	−2.25	0.19	<0.001
Sociosexuality	0.88	0.12	<0.001	LTMO	−0.26	0.11	0.015
Target orientation-long	0.28	0.09	0.001	Target orientation-long	0.38	0.06	<0.001
Sex- female	−0.91	0.30	0.003	LTMO × Target orientation	0.50	0.06	<0.001
Sociosexuality × Target orientation	−0.77	0.07	<0.001				
Sex × Target orientation	0.67	0.14	<0.001				
**Random effects**							
	ICC				ICC		
Participants	0.31				0.31		
Targets	0.26				0.26		
Pseudo-*R*^2^ (fixed/total)	0.09/0.61			Pseudo-*R*^2^ (fixed/total)	0.01/0.58		

*Note.* LTMO refers to long-term mating orientation of the participant.

#### Mate value and sexual attractiveness

To test H3 that people who perceive themselves as having high mate value would be more likely to selectively match with attractive individuals than their counterparts who have low self-perceived mate value, we ran two models for self-perceived mate value and sexual attractiveness, respectively. The results are presented in Table 4. We did not find support for either the interaction between mate value and target attractiveness or the interaction between sexual attractiveness and target attractiveness. H3 therefore did not receive sufficient support based on our pre-registered analyses.

**Table 4.** Mate value and target attractiveness on matching decision

	Mate value model				Sexual attractiveness model		
	*B*	SE	*p*		*B*	SE	*p*
**Fixed effects**							
Intercept	−2.31	0.18	<0.001	Intercept	−2.31	0.18	<0.001
Mate value	−0.05	0.10	0.597	SPSA	0.12	0.10	0.243
Target attractiveness	0.58	0.15	<0.001	Target attractiveness	0.57	0.15	<0.001
Mate value × Target attractiveness	−0.01	0.03	0.674	SPSA × Target attractiveness	0.02	0.03	0.624
**Random effects**							
	ICC				ICC		
Participants	0.33				0.32		
Targets	0.22				0.23		
Pseudo-*R*^2^ (fixed/total)	0.04/0.57			Pseudo-*R*^2^ (fixed/total)	0.05/0.57		

*Note.* SPSA refers to self-perceived sexual attractiveness of the participant.

#### Disgust sensitivity and mating decisions

To recap, we hypothesised that (1) people with higher pathogen disgust sensitivity would be more likely to selectively match with attractive individuals than their counterparts who have low pathogen disgust sensitivity (H4), (2) people with higher sexual disgust sensitivity would be less likely to match with individuals with short-term mating orientation than their counterparts who have low sexual disgust sensitivity (H5) and (3) people with higher moral disgust sensitivity would be more likely to match with individuals with long-term mating orientation than their counterparts who have low moral disgust sensitivity (H6). To test these hypotheses, we ran three separate models. The first model included pathogen disgust, target attractiveness, sex, the interaction between pathogen disgust and target attractiveness, and the interaction between sex and target attractiveness as fixed effects. The second model included sexual disgust, target orientation, sex, the interaction between sexual disgust and target orientation, and the interaction between sex and target orientation as fixed effect. The third model included moral disgust, target orientation and their interaction term as fixed effect. For all three models, we included random intercepts for participants and targets. The results are presented in Table 5. We did find a statistically significant interaction between pathogen disgust and target attractiveness; however, the direction of the interaction was contrary to what we had hypothesised. The results indicated that people with higher pathogen disgust sensitivity were less likely to selectively match with attractive individuals than their counterparts who have low pathogen disgust sensitivity. Participants with high or low pathogen disgust sensitivity did not differ in their propensity to match with the less attractive targets (*B* = −0.07, SE = 0.11, *p* = 0.547), yet high (vs. low) pathogen disgust individuals were less likely to match with targets with attractiveness ratings above the mean (*B* = −0.24, SE = 0.10, *p* = 0.023). H4 therefore did not receive support from the data. As for H5, we found support for the hypothesised interaction between sexual disgust and target orientation. People with higher (vs. lower) sexual disgust sensitivity not only were less likely to make a match decision but also were especially less likely to match with individuals with short-term mating orientation. We also found support for H6. The positive interaction between moral disgust and target orientation indicated that people with higher (vs. lower) moral disgust sensitivity would be more likely to match with individuals with long-term mating orientation.

**Table 5.** Disgust sensitivity and matching decisions

Pathogen disgust (H4)				Sexual disgust (H5)				Moral disgust (H6)			
	*B*	SE	*p*		*B*	SE	*p*		*B*	SE	*p*
**Fixed effects**				**Fixed effects**				**Fixed effects**			
Intercept	−1.83	0.21	<0.001	Intercept	−2.11	0.24	<0.001	Intercept	−2.51	0.19	<0.001
Disgust	−0.16	0.10	0.132	Disgust	−0.80	0.12	<0.001	Disgust	−0.04	0.11	0.692
Target attractiveness	0.76	0.14	<0.001	Target orientation	0.09	0.08	0.289	Target orientation	0.36	0.06	<0.001
Sex-female	−0.89	0.26	0.001	Sex-female	−1.13	0.29	<0.001	Disgust × Target orientation	0.27	0.06	<0.001
Disgust × Target attractiveness	−0.07	0.03	0.047	Disgust × Target orientation	0.48	0.07	<0.001				
Sex × Target attractiveness	−0.27	0.10	0.008	Sex × Target orientation	0.97	0.13	<0.001				
**Random effects**				**Random effects**				**Random effects**			
	ICC				ICC				ICC		
Participants	0.34			Participants	0.30			Participants	0.31		
Targets	0.19			Targets	0.25			Targets	0.26		
Pseudo-*R*^2^ (fixed/total)	0.08/0.56			Pseudo-*R*^2^ (fixed/total)	0.09/0.60			Pseudo-*R*^2^ (fixed/total)	0.01/0.58		

#### Memory for match (vs. no match) faces

To test the final hypothesis that people would have better memory for faces they chose to match with than faces they chose to pass, we ran a GLMM with recognition outcome of targets (correct vs. incorrect) as the dependent variable, match decision (match vs. not-match) and target attractiveness ratings as fixed effects. For random effects, we included random intercepts for participants and targets. The results showed that matching decision significantly predicted the correct recognition of the target, *B* = 0.46, SE = 0.08, *p* < 0.001. People had better memories for the faces they chose to match than not match, supporting H7. Target attractiveness ratings, on the other hand, were not a statistically significant predictor, *B* = −0.10, SE = 0.06, *p* = 0.128.

### Exploratory analyses

As reported in the ‘Method’ section, in addition to the normative attractiveness ratings, we also measured participants’ perception of the attractiveness of the targets and ran exploratory analyses with this measure to test H1, H3, H4 and H7. The results are reported below.

#### Sexual strategies and target attractiveness

Exploratory analysis with participant-rated target attractiveness revealed that participant-rated target attractiveness (*B* = 3.60, SE = 0.13, *p* < 0.001) but not sociosexuality (*B* = 0.18, SE = 0.15, *p* = 0.235) predicted matching decision. Interestingly, the interaction between sociosexuality and participant-rated target attractiveness was statistically significant (*B* = 0.39, SE = 0.10, *p* < 0.001), indicating that participant-rated target attractiveness rating is a stronger predictor of match decision among participants with more (vs. less) unrestricted sociosexuality, supporting H1. Neither sex (*B* = −0.49, SE = 0.30, *p* = 0.150) nor the interaction between sex and participant-rated target attractiveness (*B* = −0.11, SE = 0.18, *p* = 0.528) were statistically significant predictors of matching decision in the second model. Notably, this model with participant-rated target attractiveness (fixed effects, pseudo *R*^2^ = 0.67) explained more variance than the previous model with target attractiveness ratings from independent raters (fixed effects, pseudo *R*^2^ = 0.09).

#### Mate value and sexual attractiveness

Exploratory analyses testing H3 using participant-rated target attractiveness showed a different picture compared with the planned analyses (see Table 6). In these two models, we found support for the interaction between mate value and target attractiveness as well as the interaction between self-perceived sexual attractiveness and target attractiveness. The positive interaction terms suggested that the predictive power of target attractiveness rating on match decision was stronger among people who perceived themselves as having higher mate value or being more sexually attractive. To put it differently, people who considered themselves as high (vs. low) mate value or more (vs. less) sexually attractive were more likely to choose to match with the targets they considered attractive.

**Table 6.** Mate value and participant-rated target attractiveness on matching decision

	Mate value model				Sexual attractiveness model		
	*B*	SE	*p*		*B*	SE	*p*
**Fixed effects**							
Intercept	−3.75	0.15	<0.001	Intercept	−3.74	0.15	<0.001
Mate value	−0.23	0.15	0.121	SPSA	−0.02	0.14	0.912
Participant-rated target attractiveness	3.61	0.10	<0.001	Participant-rated target attractiveness	3.61	0.10	<0.001
Mate value × Participant-rated target attractiveness	0.25	0.08	0.002	SPSA × Participant-rated target attractiveness	0.27	0.08	0.001
**Random effects**							
	ICC				ICC		
Participants	0.52				0.47		
Targets	0.004				0.005		
Pseudo-*R*^2^ (fixed/total)	0.66/0.84			Pseudo-*R*^2^ (fixed/total)	0.66/0.84		

*Note.* SPSA refers to self-perceived sexual attractiveness of the participant.

#### Pathogen disgust sensitivity and mating decisions

Using participant-rated target attractiveness rating, we ran another model to test H4 that participants with higher (vs. lower) pathogen disgust sensitivity would be more likely to selectively match with attractive individuals. In this model, we also added sex as well as the interaction between sex and participant-rated target attractiveness as control. We did not find support for the hypothesised interaction between pathogen disgust sensitivity and participant-rated target attractiveness (*B* = −0.02, SE = 0.09, *p* = 0.808). Taken together, H4 was not supported by either test.

#### Memory for match (vs. no match) faces

As exploratory analyses reported above showed that participant-rated target attractiveness ratings outperformed normative target attractiveness ratings in predicting participants’ matching decisions, we ran a second model with participant-rated target attractiveness instead of the normative target attractiveness rating in the model. Matching decision remained a significant predictor of the correct recognition of the target, *B* = 0.26, SE = 0.09, *p* = 0.005. Participant-rated target attractiveness also turned out to be a significant positive predictor, *B* = 0.19, SE = 0.04, *p* < 0.001.

## Discussion

In the current study, we examined how theoretically relevant individual differences interacted with target properties such as target attractiveness and mating goal to predict matching decisions in a mock online dating task. The results showed patterns of assortative mating regarding mating goals and self-perceived mate value. Further, moral disgust and sexual disgust interacted with the target's mating goal to predict matching decisions per our hypotheses. However, we did not find support for the moderating effect of pathogen disgust. Finally, our novel hypothesis that people would have worse memories for the faces they choose to not match received support. In the following sections, we discuss the results and their theoretical implications in detail.

### Assortative mating

Assortative mating is ubiquitous (for a recent meta-analysis, see Horwitz et al., 2023). People find partners that are similar to themselves in terms of political and religious attitudes, educational attainment (e.g. Eika et al., 2019; Gonggrijp et al., 2023; Mare, 1991) and psychological (e.g. personality, De La Mare & Lee, 2023; Zhang et al., 2022) and anthropometric traits (e.g. height, Pisanski et al., 2022; race, Ranzini et al., 2022).

In Zhang et al. (2022), the authors reported that people with a more unrestricted sociosexuality (i.e. greater short-term mating orientation) emphasise less a potential partner's commitment compared with people who are more sociosexually restricted. On the other hand, people who pursue a long-term mating strategy pay more attention to commitment than people who are less concerned with long-term mating. Moving beyond self-reported preference, our study showed similar behavioural results using a mock online dating task. Participants tend to match with individuals who share their relationship goals.

Even though our planned analyses using normative target attractiveness ratings did not find support for the assortative mating pattern regarding self-perceived mate value and targets’ attractiveness, using participant-rated attractiveness, we did find that the likelihood of matching with the targets one was attracted to is greater among people who perceived themselves as having higher mate value or sexual attractiveness than people with low self-perceived mate value or sexual attractiveness. This might be a cost–benefit analysis done taking into consideration how one is perceived by potential mates in the market, which could be one of the mechanisms underlying various assortative mating patterns such as education attainment. That is, people would consider their own value as a mate while deciding whether or not they wanted to initiate an interaction and would be more likely to match when they believed that they were attractive in the eyes of their potential mates.

Regarding the discrepant results between normative target attractiveness ratings and participant-rated attractiveness, one possible explanation is that the normative rating by the independent sample (i.e. a group average) does not correspond well with how specific individuals perceive the targets. That is, there is heterogeneity among participants’ attractiveness ratings of the same target, evidenced by the fact that the normative attractiveness ratings explained only 4% of the variance in participant-rated attractiveness. As the saying goes, beauty is in the eyes of the beholder. A second explanation is that the target attractiveness ratings by participants in the current experiment were not only the perceived physical attractiveness of the targets but an overall attractiveness judgment taking into other types of information. To see if this conjecture is consistent with our data, we ran exploratory LMM analyses on participant-rated target attractiveness with their sexual strategies and target's mating orientation, as well as the interaction terms as fixed effects. The results showed that on average targets with long-term orientation were perceived as more attractive. Moreover, this effect was moderated by participants’ own sexual strategies. Long-term-oriented targets were not perceived as more attractive or even were perceived as less attractive than short-term-oriented targets by participants with greater short-term mating orientation (for the model outputs, see Appendix Table A1). On the other hand, long-term-oriented targets were perceived as more attractive by participants with greater (vs. lower) long-term orientation. Thus, the idea that target attractiveness ratings were influenced by more than physical aspects received support. Note that, the two explanations are not mutually exclusive and may well work in parallel.

### Disgust sensitivity and matching decisions

Contrary to our hypothesis, we did not find support that people with high pathogen disgust would selectively match with attractive individuals. In fact, per the planned analysis, we found that people with high pathogen disgust sensitivity were ‘pickier’ and less likely to match with the relatively more attractive targets compared with their low pathogen disgust peers. This result seems to be inconsistent with the self-report measures from Zhang et al. (2022), in which the positive association between pathogen disgust and preference for physical attractiveness was observed in three samples from two countries. Given the recent null finding regarding pathogen disgust and preference for sexual dimorphic or symmetric faces (Tybur, Fan et al., 2022), one possibility is that there is no real relationship between the two psychological measures. Yet this possibility is also at odds with the results which showed an opposite effect to that expected. Another explanation is that the stimuli used in the current experiment are ill-suited to test H3. Although the photos in the OFD vary in their attractiveness, they have one shortcoming when it comes to the research on pathogen disgust and infection risk. That is, none of the photos showed obvious signs of low hygiene or infection cues. Perceived physical attractiveness in real-life settings is not only determined by sexually dimorphic features or symmetricity of the faces but also by the varying bodily hygiene and skin conditions that signal infection risks such redness or blemishes (Kowal et al., 2022). Pathogen disgust, as the proposed behavioural immune system, should be more sensitive to pathogen cues (e.g. pathogen-related odours (Tybur, Croijmans et al., 2022) and facial blemishes (van Leeuwen & Jaeger, 2022)) than the supposedly indirect cues of immune functioning such as facial structures (Tybur, Fan et al., 2022).

Regarding sexual disgust sensitivity, we found that people with high (vs. low) sexual disgust were less likely to match with the targets, especially with the targets showing a short-term mating goal. This result is consistent with previous research showing a robust negative correlation between short-term mating and sexual disgust using trait measures (e.g. Al-Shawaf et al., 2015; O'Shea et al., 2019; Zhang et al., 2022), which we also observed in the current study. More importantly, the interaction between sexual disgust and the target's mating goal indicated that sexual disgust functions as a behavioural avoidance mechanism that inhibits more risky sexual behaviour such as short-term mating but has less effect inhibiting the pursuit of safer, long-term-oriented relationships.

Moral disgust is disgust elicited by social transgressions including non-normative behaviours and even antisocial conduct and functions to promote social coordination (Tybur et al., 2009, 2013). As discussed by Zhang et al. (2022), even though both types of mating behaviours (short-term and long-term) are socially approved (in a sample from the Netherlands), people on average perceive long-term mating as more approved by society. Therefore, one can expect that individuals who have a higher moral disgust sensitivity could be more compliant with social norms, showing a greater preference for long-term mating. They indeed found that individuals with higher (vs. lower) moral disgust sensitivity not only prefer long-term mating themselves, but also placed more value on mate commitment, preferring potential mates that are pursuing a long-term mating strategy. In the current study, we found similar results with behavioural data. On average, people are more likely to match with long-term-oriented individuals, showing a general preference for long-term mating. Further people with higher moral disgust, compared with their low moral disgust peers, were more likely to match with long-term-oriented individuals.

Taken together, the results from the current study offer further support for the adaptationist functional perspective of disgust, showing that sexual disgust and moral disgust predicted online dating behaviours consistent with theory-driven hypotheses. However, the relationship between pathogen disgust and preference for potential mates’ attractiveness remains unclear.

### The role of memory in mating psychology

Inspired by the research on directed forgetting showing that compared with stimuli designated for remembering, individuals often exhibit poorer memory for stimuli instructed to be forgotten (e.g. Basden et al., 1993; Corenblum et al., 2020; Hauswald & Kissler, 2008; Metzger, 2011; Thompson et al., 2011), we examined if people would have better memories for the faces they choose to match than the faces they choose to not match. As expected, after a 24 h retention interval, people had better performances recognising the faces they decided to match with than the faces they decided to not match. This effect held when controlling either the normative target attractiveness rating or the participant-rated target attractiveness. Given that various individual differences and contextual information may influence an individual's mating decisions, they may indirectly influence the perceived mating pools (Crosby et al., 2021) among individuals with various traits via memory. In Crosby et al. (2021), participants with higher (vs. lower) sexual disgust perceived their mating pool to be smaller. In our study, we showed that high sexual disgust individuals, compared with their low sexual disgust counterparts, were less likely to choose to match with potential mates and that targets who are not considered ‘datable’ at one moment may be easily forgotten and not considered as one potential mate by individuals at later times. Our results therefore possibly offer one mechanistic explanation on how individuals’ preferences and motivations shape (or distort) their perceived mating pools. Distorted mating pool perception could contribute to negative mental health such as loneliness and problematic coping, with the extreme case being the involuntary celibacy community (e.g. Sparks et al., 2023).

### Limitations and future directions

First and foremost, the faces from the Oslo Face Database (Chelnokova et al., 2014) are from university students, who are mostly white, which limits the generalisability of the findings and the ecological validity of the experimental setup. Even though online dating is not limited to young adults (Bonilla-Zorita et al., 2021), much research on this topic, including the current effort, has relied on young adult samples (e.g. De La Mare & Lee, 2023; Ranzini et al., 2022; Sumter & Vandenbosch, 2019). Future research could include more age-representative samples and profiles while studying online dating decision-making. The lack of ethnic diversity in the current experiment materials may have resulted in the underestimation of the effects, given that the UK is a multi-ethnic country (Catney, 2020). Second, as discussed earlier, the Oslo Face Database itself may not be suited to examining the relationship between pathogen disgust and mating preference because of the lack of infection cues. Future studies could, however, add infection cues to the photos (e.g. with software such as Photoshop) while designing the experiments.

Finally, the sample included in the current study mostly consisted of heterosexual individuals. However, evidence suggests that mate preferences differ between heterosexual and sexual/gender minorities such as gay men (e.g. Cordes et al., 2021; Štěrbová et al., 2021). Further, sexual norms (e.g. Valentova et al., 2020) and mating dynamics (e.g. Ying et al., 2023) differ between heterosexual communities and sexual minorities. For example, in Valentova et al. (2020), the prevalence of consensual non-monogamy was higher among sexual/gender minorities (bisexual women, 17.1%; bisexual men, 22.6%; lesbian, 7.1%; gay men, 18.8%) than heterosexual men (8.3%) and women (4.7%). In a simulation study on sex and sexual orientation differences in short-term mating, Ying et al. (2023) showed that when male (vs. female) agents were set to have a stronger preference for short-term mating, gay men had a higher average number while lesbians had a lower number of sexual experiences and mates compared with heterosexual men and women. Beyond increasing representation and inclusivity in research, it could prove to be valuable in disentangling the effect of hypothesised innate evolved processes on mate choice and preference from cultural and normative influences to test hypotheses in samples of sexual minorities and compare the results with heterosexual samples.

## Conclusion

In a mock online dating task, we found behavioural evidence for assortative mating regarding sexual strategies. People with greater short-term (long-term) mating orientation were more likely to match with short-term (long-term) oriented targets. Further, people who perceived themselves as having higher (v. lower) mate value or being more (vs. less) sexually attractive were more likely to match with individuals they considered as attractive. Moral disgust sensitivity and sexual disgust sensitivity negatively predicted matching with short-term-oriented targets, consistent with the evolution-informed hypotheses. Contrary to our hypothesis, people with higher (vs. lower) pathogen disgust sensitivity were less likely to match with more attractive targets. Finally, people have better memories of the faces they choose to match than to not match, which could underlie the individual differences in perceived mating pools.

## Research transparency and reproducibility

All data and analysis scripts are posted at https://osf.io/s6fp4/.

## Appendix

**Table A1.** Sexual strategy and target's mating goal on perceived attractiveness

	SOI-R model				LTMO model		
	*B*	SE	*p*		*B*	SE	*p*
**Fixed effects**							
Intercept	2.84	0.10	<0.001	Intercept	2.85	0.10	<0.001
Sociosexuality	0.14	0.05	0.012	LTMO	0.00	0.05	0.990
Target orientation-long	0.08	0.02	<0.001	Target orientation-long	0.08	0.02	<0.001
Sociosexuality × Target orientation	−0.10	0.02	<0.001	LTMO × Target orientation	0.05	0.02	0.012
**Random effects**							
	ICC				ICC		
Participants	0.33				0.33		
Targets	0.27				0.27		
Pseudo-*R*^2^ (fixed/total)	0.01/0.60			Pseudo-*R*^2^ (fixed/total)	0.001/0.60		
